# HIV-Related Medical Admissions to a South African District Hospital Remain Frequent Despite Effective Antiretroviral Therapy Scale-Up

**DOI:** 10.1097/MD.0000000000002269

**Published:** 2015-12-18

**Authors:** Graeme Meintjes, Andrew D. Kerkhoff, Rosie Burton, Charlotte Schutz, Andrew Boulle, Gavin Van Wyk, Liz Blumenthal, Mark P. Nicol, Stephen D. Lawn

**Affiliations:** From the Clinical Infectious Diseases Research Initiative, Institute of Infectious Disease and Molecular Medicine (GM, CS, LB); Department of Medicine, Faculty of Health Sciences, University of Cape Town (GM, RB, CS, SDL); Department of Medicine, Khayelitsha District Hospital, South Africa (GM, RB); Department of Medicine, Imperial College London, London, UK (GM); The Desmond Tutu HIV Centre, Institute of Infectious Disease and Molecular Medicine, Faculty of Health Sciences, University of Cape Town, South Africa (ADK, SDL); Department of Medicine, University of California San Francisco School of Medicine, San Francisco, CA, USA (ADK); Department of Global Health, Academic Medical Center, Amsterdam Institute for Global Health and Development, University of Amsterdam, Amsterdam, The Netherlands (ADK); School of Public Health and Family Medicine, Faculty of Health Sciences, University of Cape Town (AB); Health Impact Assessment Directorate, Western Cape Department of Health (AB); Institute of Infectious Disease and Molecular Medicine, Faculty of Health Sciences, University of Cape Town (AB); Department of Medicine, Mitchells Plain Hospital (GVW); Division of Medical Microbiology, Faculty of Health Sciences, University of Cape Town (MPN); National Health Laboratory Service, South Africa (MPN); and Department of Clinical Research, Faculty of Infectious and Tropical Diseases, London School of Hygiene and Tropical Medicine, London, UK (SDL).

## Abstract

The public sector scale-up of antiretroviral therapy (ART) in South Africa commenced in 2004. We aimed to describe the hospital-level disease burden and factors contributing to morbidity and mortality among hospitalized HIV-positive patients in the era of widespread ART availability.

Between June 2012 and October 2013, unselected patients admitted to medical wards at a public sector district hospital in Cape Town were enrolled in this cross-sectional study with prospective follow-up. HIV testing was systematically offered and HIV-infected patients were systematically screened for TB. The spectrum of admission diagnoses among HIV-positive patients was documented, vital status at 90 and 180 days ascertained and factors independently associated with death determined.

Among 1018 medical admissions, HIV status was ascertained in 99.5%: 60.1% (n = 609) were HIV-positive and 96.1% (n = 585) were enrolled. Of these, 84.4% were aware of their HIV-positive status before admission. ART status was naive in 35.7%, current in 45.0%, and interrupted in 19.3%. The most frequent primary clinical diagnoses were newly diagnosed TB (n = 196, 33.5%), other bacterial infection (n = 100, 17.1%), and acquired immunodeficiency syndrome (AIDS)-defining illnesses other than TB (n = 64, 10.9%). By 90 days follow-up, 175 (29.9%) required readmission and 78 (13.3%) died. Commonest causes of death were TB (37.2%) and other AIDS-defining illnesses (24.4%). Independent predictors of mortality were AIDS-defining illnesses other than TB, low hemoglobin, and impaired renal function.

HIV still accounts for nearly two-thirds of medical admissions in this South African hospital and is associated with high mortality. Strategies to improve linkage to care, ART adherence/retention and TB prevention are key to reducing HIV-related hospitalizations in this setting.

## INTRODUCTION

South Africa has the largest human immunodeficiency virus (HIV) epidemic in the world with an estimated 6.4 million people living with HIV infection.^1^ The public sector antiretroviral (ART) programme was launched in 2004.^2^ Over the past decade there has been an unprecedented scale-up of the programme with more than 2.6 million people having initiated ART.^3,4^ ART is now available free of charge at 3736 public health facilities across South Africa to those eligible based on clinical and cluster of differentiation (CD)4 count criteria. HIV-related mortality has decreased^5^ and life expectancy has increased to approximately 80% of normal life expectancy.^6^ It is estimated that 2.2 million deaths will have been averted by 2016.^7^

In developed countries, the availability of triple-drug ART in the mid-1990s heralded dramatic reductions in acquired immunodeficiency syndrome (AIDS)-defining illnesses and hospital admissions for HIV-related opportunistic infections.^8–14^ In South Africa, prior to widespread ART availability, HIV/AIDS accounted for approximately 50% of medical ward admissions in public sector hospitals.^15,16^ A recent systematic review and meta-analysis has summarized data on causes of hospital admission among children and adults living with HIV globally: AIDS-related illnesses (including tuberculosis [TB]) and bacterial infections were the 2 commonest causes of adult HIV admissions in all geographical regions and the most common causes of hospital mortality.^17^ However, a decade into widespread ART scale-up, little is known about the impact of the ART programme on adult HIV-related hospitalizations and outcomes at the level of public sector hospitals in South Africa.

Thus, we sought to define the hospital-level epidemiology of HIV infection a decade after the launch of the world's largest public sector ART programme. We aimed to determine the proportion of hospital admissions related to HIV infection, admission diagnoses, to describe patients’ prior access to HIV treatment services, and to determine the associated mortality and factors that contributed to mortality. In the discussion, based on our findings, we explore reasons why in the presence of ART availability a substantial number of admissions and deaths continue to occur.

## METHODS

### Setting and Patients

This cross-sectional study with prospective follow-up was conducted between 6th June 2012 and 4th October 2013, at G.F. Jooste Hospital. This 200-bed adult public sector district hospital is located in the Western Cape province of South Africa and serves township communities of around 1.3 million people. In these communities, the HIV seroprevalence approximates to the national estimate; the antenatal HIV seroprevalence in Khayelitsha in 2013 was 34.4%.^18^ The vast majority of people living in these communities rely on the public health system for hospital admission. ART has been freely available in the public sector since April 2004.^2^ During the initial study period (June 2012–April 2013), patients were eligible for ART if their CD4 count was <200 cells/μL (or <350 cells/μL if they were pregnant or had TB) or if they had any World Health Organization (WHO) stage 4 illness. The criteria expanded in April 2013 to include all with CD4 counts ≤350 cells/μL or WHO stages 3 or 4 disease. By December 2013, it is estimated that 41,806 adult patients were on ART in 20 public sector clinics in the referral area of this hospital (Western Cape Government, unpublished). ART coverage was regarded as above average compared to national coverage in these communities.^19^

The primary aim of the parent study was to assess the diagnostic yield of performing routine microbiological screening for TB among unselected HIV-infected patients admitted to the medical wards. The sample size was set in order to determine the prevalence of newly diagnosed microbiologically confirmed TB with adequate precision (95% confidence interval of +/−5%) in the parent study.^20^ Here, we report a secondary analysis focused on overall admission diagnoses and outcomes. Adult patients aged ≥18 years were recruited on 4 days of each week from the 2 medical wards. To avoid selection bias, on recruitment days the study coordinator recorded from the ward register all medical admissions in a 24-hour period. All patients with previously negative or undocumented HIV status were offered HIV counseling and testing using 2 rapid tests: ABON HIV1/2/0/Tri-line HIV rapid test (Alere Inc., Waltham, MA) and Determine HIV-1/2 rapid test (Alere Inc.). All patients testing HIV-positive and all those documented previously to be HIV-positive were invited to participate. The study was approved by the Human Research Ethics Committees of the University of Cape Town and the London School of Hygiene and Tropical Medicine. All participants provided written informed consent. This study is reported in conformity with the Strengthening the Reporting of Observational Studies in Epidemiology (STROBE) statement checklist for observational studies.

### Procedures

Demographic and clinical details were recorded on standardized forms. The research team systematically obtained sputum, urine, and blood specimens for TB investigations in the first 24 hours of admission.^20^ Numerous additional samples for TB investigations were obtained by ward teams during the admission period. Results of TB tests conducted up to 1 year prior to admission at primary care clinics were obtained from the National Health Laboratory Service (NHLS) electronic records. The ward teams undertook other investigations as directed by the clinical scenario. Biochemistry, hematology, and microbiological tests were conducted at the NHLS.

### Data Acquisition and Definitions

The admission diagnoses were extracted from patients’ medical records, and this was supplemented with additional data from the NHLS laboratory results database. Two investigators (GM and ADK) reviewed all the diagnostic information obtained and assigned each participant into one of the following mutually exclusive, prespecified categories for their primary clinical diagnosis by consensus: new TB diagnosis (took priority over other diagnoses); clinical deterioration of TB cases during treatment (included the following underlying causes: poor adherence, drug resistant TB, TB-associated immune reconstitution inflammatory syndrome, hemoptysis, and pneumothorax); AIDS-defining illnesses other than TB (included opportunistic infections and AIDS-related malignancies); noncommunicable diseases (NCDs, included non-AIDS cancer, diabetes, hypertensive complications, heart failure, and asthma); other bacterial infection (including pneumonia and dysentery); venous thromboembolism; drug-related (adverse effects or overdose); psychiatric illness; major organ dysfunction (MOD) not attributable to one of the above categories (included renal and liver failure, bone marrow dysfunction, seizures, and stroke); or other. In a separate categorization, all patients with a neurological diagnosis across all the primary clinical diagnoses were identified.

A new TB diagnosis was defined by detection of *Mycobacterium tuberculosis* from any clinical sample using culture or Xpert MTB/RIF or a physician's decision to start of full course of empiric TB treatment. This category (new TB diagnosis) included not only all diagnoses made during the admission period, but also diagnoses within 7 days prior to the index admission (at any hospital or clinic).

Patients’ ART status was classified (naive, current, or interrupted) on the day of admission. Vital status 90 and 180 days after the admission date was determined using patient case notes and the medical ward register in addition to 5 electronic health record databases that were searched systematically by hospital folder numbers as well using different combinations of a patients’ identifying information. Additional patient deaths were ascertained by cross-referencing patient identification numbers with death certificates through the South African Department of Home Affairs database. The multiple databases were cross-referenced for consistency in patient outcomes and any discrepancies were resolved. For patients in whom vital status could not be ascertained from one of the 8 data sources, the outcome was defined as “could not be traced.” An HIV viral load <400 copies/mL during admission was regarded as virologically suppressed. Estimated glomerular filtration rate was calculated using the Modification of Diet in Renal Disease study equation.^21^

### Data Analysis

Medians were compared using either Wilcoxon rank-sum tests or Kruskal–Wallis tests, and means were compared using unpaired *t*-tests. Chi-squared and Fisher exact tests were used to compare proportions. The main survival analyses that we present focused on 90-day mortality, since deaths within this time period most likely related to the index admission diagnosis and follow-up data were very complete up to this time-point (∼95%). Patients with unknown 90-day outcomes were censored on the date they were last seen alive in a health care setting. Kaplan–Meier survival analyses compared 90-day mortality based on primary clinical diagnosis, ART category, and virological suppression; the log-rank test was used to compare groups. A Cox proportional hazards model was constructed to identify independent predictors of mortality. All variables in the univariable model meeting a cut-off of *P* ≤ 0.1 and age (an a priori risk factor) were included in the multivariable model. Schoenfeld's global test was used to test the proportional hazards assumption.^22^ All statistical tests were 2-sided at α = 0.05.

## RESULTS

**FIGURE 1:**
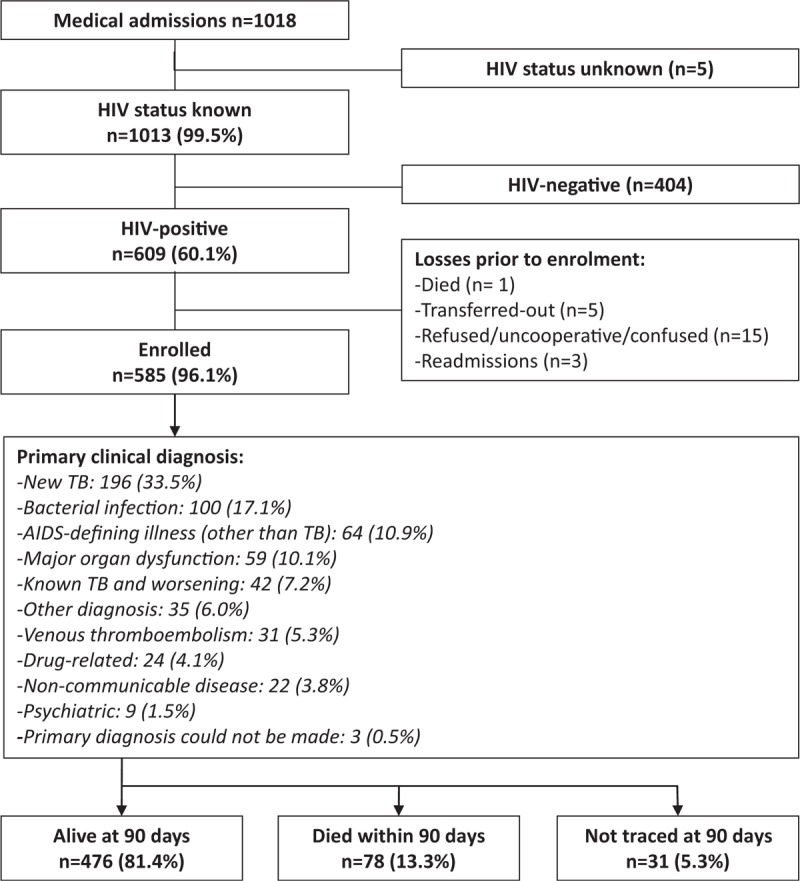
Study profile: this flow diagram shows the numbers of patients screened for the study, reasons for exclusion, number enrolled, primary clinical diagnoses, and 90-day outcomes.

The HIV status of 1013 of 1018 (99.5%) unselected new admissions to the adult medical wards was ascertained. Of the 609 patients (60.1%) with a positive HIV test, 585 (96.1%) were enrolled (Fig. 1).

### Reasons for Admission

The most frequent primary clinical diagnosis was newly diagnosed TB (n = 196, 33.5%) followed by other bacterial infection, AIDS-defining illnesses other than TB, MOD, and worsening of known TB (Fig. 1). There were 88 patients (15.0%) with neurological diagnoses, most frequently TB meningitis (n = 27) and cryptococcal meningitis (n = 18).

A total of 44% of admissions (n = 255) were directly related to TB: 196 with a new TB diagnosis, 42 with clinical deterioration during treatment for TB, and 17 with an adverse reaction to TB treatment (16 of these were drug-induced liver injury). An additional 78 patients (13.3%) were receiving TB treatment at admission, but the reason for index admission was another diagnosis such as other bacterial infection or AIDS-defining illness. Thus, 56.9% (n = 333) of all patients had either a primary or secondary diagnosis of TB. Of 196 patients with a new TB diagnosis, 160 (81.6%) were confirmed by either culture of *M. tuberculosis* or Xpert MTB/RIF on a range of clinical samples. Of the 36 patients without a confirmed microbiological diagnosis, 14 were diagnosed with TB meningitis based on clinical findings and cerebrospinal fluid analysis, and most of the others were started on empiric TB treatment in primary care immediately prior to admission.

### HIV Diagnosis and ART Exposure

Of the 585 HIV-infected patients, 93 (15.9%) had new HIV diagnoses made during the index admission and their median CD4 count was 73 cells/μL (interquartile range [IQR] 40–188). Overall, 209 (35.7%) patients were ART-naive; 376 (64.3%) were ART-experienced with 263 currently receiving ART (45.0%) and 113 (19.3%) having interrupted therapy. Most baseline characteristics did not differ by ART exposure (Table 1). However, the median CD4 count was lowest in those who had interrupted (median 71 cells/μL; and 62.8% of these patients had CD4 < 100 cells/μL) compared with those who were ART-naive and those currently on ART (Table 1**)**. The median ART duration for patients currently receiving ART was 1.1 years (IQR = 0.2–2.9), 26.5% had started ART within the preceding 90 days. Virological suppression was not significantly associated with duration of ART, the proportion virologically suppressed among those on ART for <6 months was 49.5%, for 6 to 12 months 58.6% and for those ≥12 months on ART 58.0% (*P* = 0.40). A higher proportion of patients who had interrupted ART had been on second-line ART compared with those currently on ART (Table 1).

**TABLE 1:**
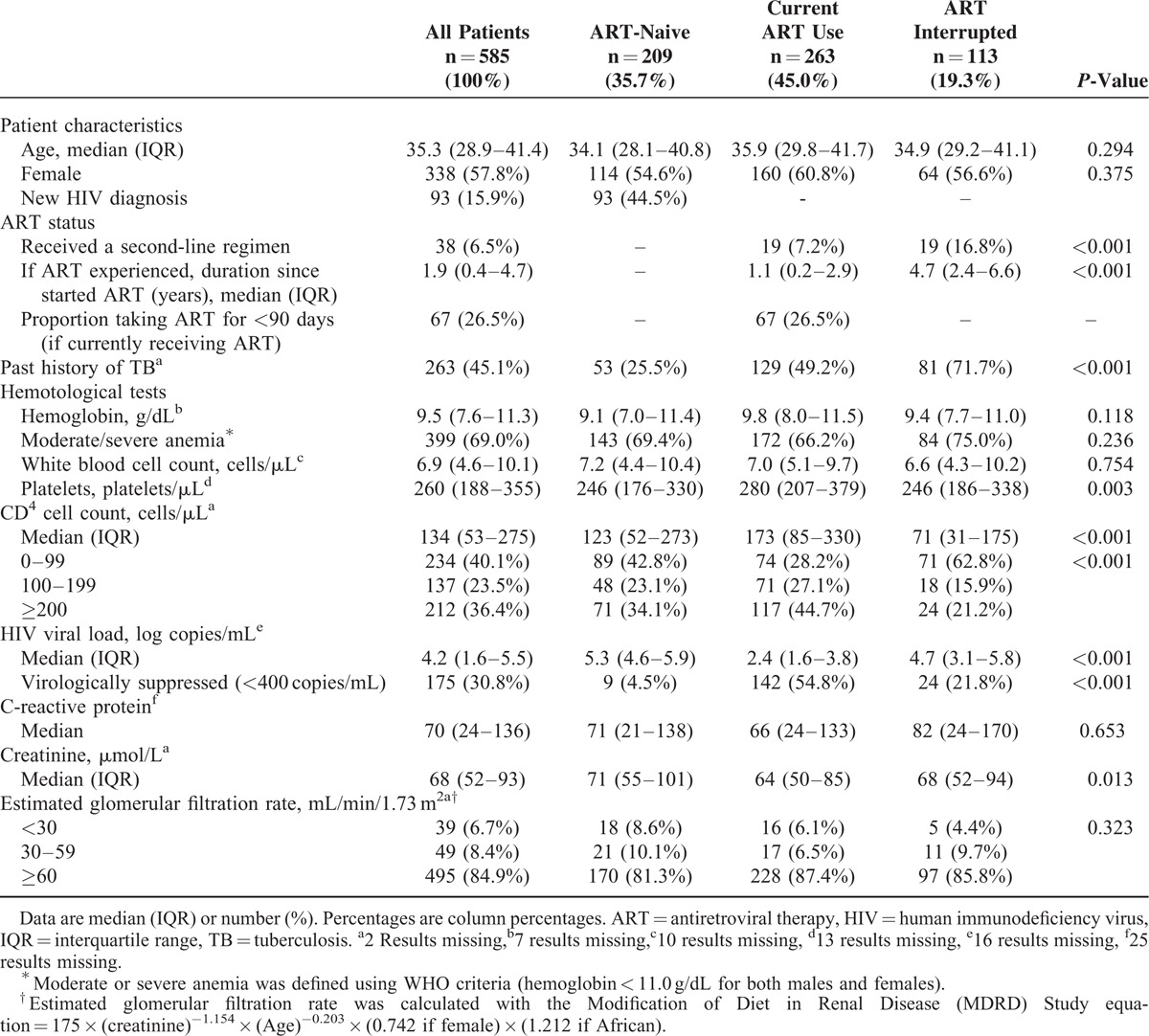
Baseline Characteristics for 585 Patients With HIV Infection Admitted to Medical Ward

### Cascade of Care

**FIGURE 2:**
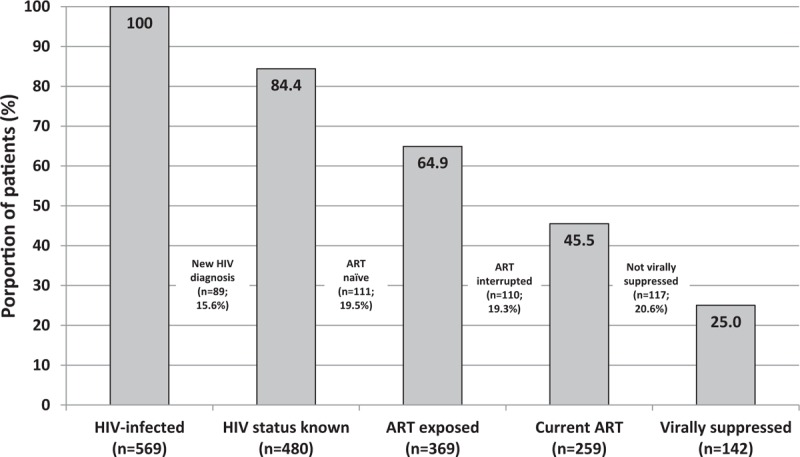
Cascade of care at G.F. Jooste Hospital among all HIV-infected patients with a viral load result available (n = 569). These categories relate to patients’ status in the care cascade on the day of admission. “HIV status known” refers to patients who were diagnosed with HIV prior to the index admission.

Of the 585 patients, HIV viral load results were available for 569 (97.3%) and these data were used to help classify these patients in the cascade of HIV care (Fig. 2). There was a substantial fall-off at each step of the cascade of HIV care pathway. Overall, just 25.0% (n = 142) were currently taking ART and were virologically suppressed.

### Patients Currently Taking ART and Virologically Suppressed

These 142 patients who were virologically suppressed represented 54.8% of those currently on ART and who had a viral load result. The median CD4 count in these patients was 251 cells/μL (IQR 136–449), and their median duration on ART was 1.1 years (IQR 0.3–3.6). The most frequent reasons for admission in these patients were newly diagnosed TB (23.2%), other bacterial infection (17.6%), MOD (15.5%), and venous thromboembolism (10.6%). Noninfectious reasons for admission tended to be more frequent in these patients compared to those who were currently on ART but not virologically suppressed (Supplementary Table 1).

### Outcomes and Predictors of Mortality

In-hospital mortality at G.F. Jooste Hospital during the index admission was 5.3% (n = 31). At 90 days of follow-up, 78 patients had died (13.3%) and 31 (5.3%) could not be traced. The 90-day cumulative mortality varied according to primary clinical diagnosis (Supplementary Table 2): for patients with newly diagnosed TB it was 10.7%, for those with known TB deteriorating on TB treatment it was 9.5%, for those with other AIDS-defining illnesses it was 29.7%, and for those with NCDs it was 27.3%.

**FIGURE 3:**
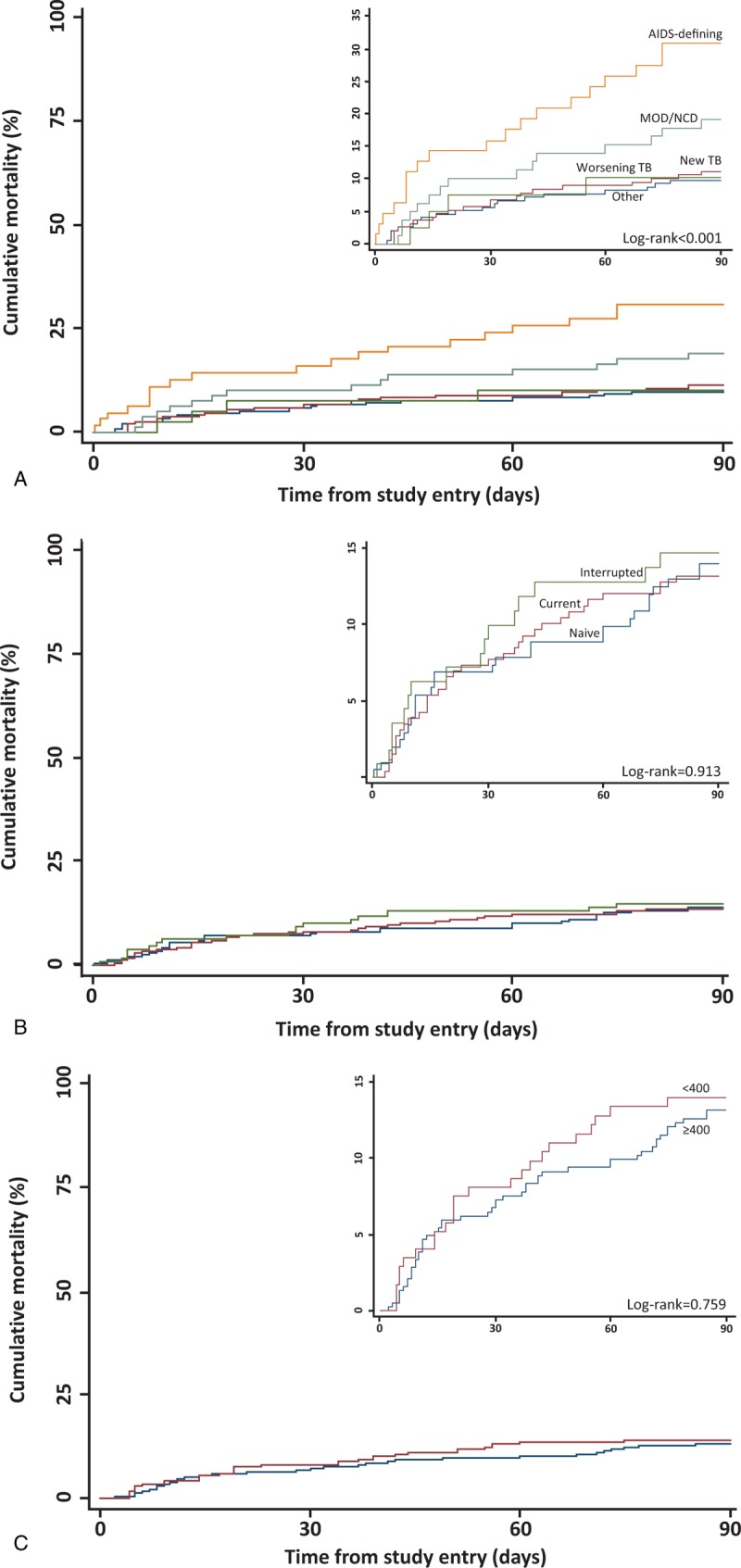
(A) Kaplan–Meier 90-day mortality estimate among all HIV-infected patients by primary diagnosis category. (B) Kaplan–Meier 90-day mortality estimate among all HIV-infected patients by ART status at the time of admission. (C) Kaplan–Meier 90-day mortality estimate among all HIV-infected patients by viral load result (viral load performed on day of admission). AIDS-defining = acquired immunodeficiency syndrome-defining illnesses other than TB, HIV = human immunodeficiency virus, MOD = major organ dysfunction, NCD = noncommunicable disease, TB = tuberculosis, Worsening TB = clinical deterioration of TB cases during treatment.

Figure 3A shows Kaplan–Meier analysis of 90-day mortality, demonstrating significantly higher mortality in those with a non-TB AIDS-defining diagnosis compared with other diagnoses (*P* < 0.001). Among patients with a neurological diagnosis 10/88 (11.4%) died by 90 days and of those with an AIDS-related neurological diagnosis 6/24 (25.0%) died by 90 days. In Kaplan–Meier analyses, there was no significant difference in mortality according to ART-status at the time of admission (Fig. 3B, *P* = 0.913) or by HIV viral load suppression status on the day of admission (Fig. 3C, *P* = 0.759). Of the 78 deaths by 90 days, 37.2% were attributable to TB (new or deteriorating TB or adverse reaction to TB treatment), 24.4% to AIDS-defining conditions other than TB, and 19.2% to major organ dysfunction or NCDs (Supplementary Figure).

Factors associated with 90-day mortality were analyzed in a Cox regression model (Table 2). Having a diagnosis of an AIDS-defining illness other than TB, lower hemoglobin levels, and lower glomerular filtration rates were independently associated with mortality. CD4 count did not independently predict mortality.

**TABLE 2:**
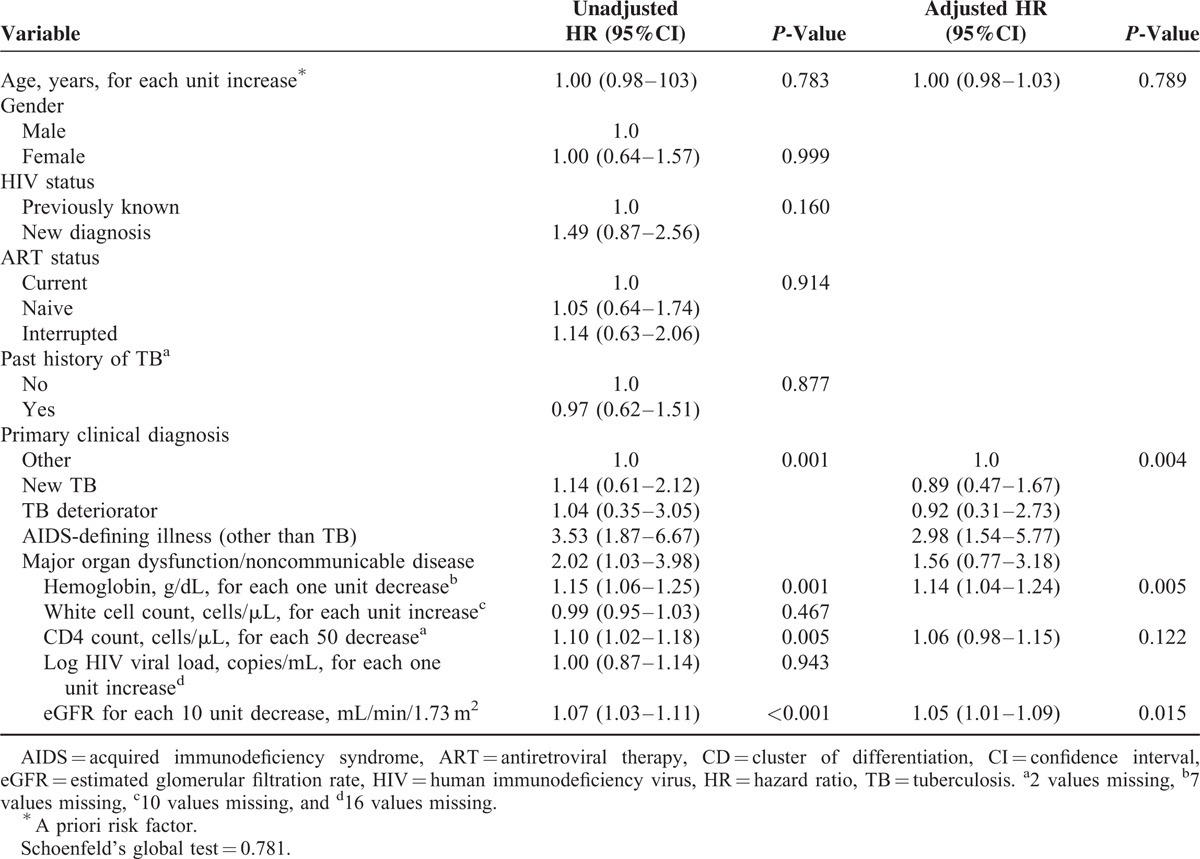
Cox Regression of Risk Factors for 90-day Mortality Among all Patients (n = 572 in Final Model With Complete Results)

Patients frequently required hospital readmission following discharge: 29.9% (n = 175) were readmitted to hospital at least once within 90 days of the index admission. Of patients currently on ART, 30.8% (n = 81) required readmission compared to 30.1% (n = 63) and 27.4% (n = 31) of those who were not on ART or had interrupted ART, respectively (*P* = 0.81). A similar proportion of virologically suppressed patients were readmitted compared to those without virological suppression but receiving ART (28.9% vs 31.6%; *P* = 0.63).

### Outcomes at 180 Days

By 180 days, 104 (17.8%) had died and 50 (8.6%) could not be traced. The main primary clinical diagnoses among those who died were TB-related (n = 41, 39.4%), other AIDS-defining illnesses (n = 22, 21.2%), and major organ dysfunction/NCD (n = 20, 19.2%). In the Cox regression model, the same factors were also independently associated with 180-day mortality: having a diagnosis of an AIDS-defining illness other than TB, lower hemoglobin levels, and lower glomerular filtration rates (data not shown).

## DISCUSSION

The public health benefit of ART in South Africa has been enormous with greatly increased life expectancy.^5,6^ Nonetheless, despite widespread ART availability, the burden of HIV-associated morbidity and mortality at public sector hospitals remains substantial with cost and resource implications.^23^ Over 60% of medical admissions at our hospital were HIV-related. Mortality at 90 days was 13.3% among HIV-infected patients; likely an underestimate as approximately 5% could not be traced.^24,25^ One-third required readmission within 90 days, reflecting their clinical complexity and resulting in additional utilization of scarce health care resources. A better understanding of reasons for this ongoing high number of HIV-related admissions is needed for designing interventions that address contributing comorbidities and health system factors.

At this same hospital in 2003 (prior to widespread ART scale-up), 43% of medical admissions were confirmed or suspected to be HIV-infected.^15^ Although HIV testing was not universal during this earlier study (limiting comparability), it suggests that despite increasing ART availability in communities surrounding the hospital for a decade, the proportion of HIV-related admissions has not decreased. This contrasts experiences in Europe and North America where HIV became a condition largely managed in outpatient settings upon introduction of triple-drug ART in the mid-1990s.^10,12–14^ A number of explanations for this difference are plausible, including health system issues and the different spectrum of opportunistic infections and other morbidities affecting HIV-infected individuals. The increasing absolute number of patients progressing to advanced HIV-related immunosuppression in the hospital's referral area over the period likely also contributed.

Several “cracks” in the cascade of care contributed to the majority of admitted patients not being virologically suppressed (Fig. 2). In 15.6% of patients, their HIV diagnosis had not been made prior to admission, demonstrating that HIV testing is not yet detecting all those infected before the onset of serious HIV-related morbidity. The majority of HIV diagnoses had been previously been made, indicating that these patients had at least initial contact with outpatient healthcare services. Of patients with an HIV diagnosis made prior to admission (n = 480), 23.1% were ART naive. There are several possible reasons for this: including recent HIV diagnosis, ART refusal, ART initiation delayed, or ineligibility based on contemporary guidelines at HIV diagnosis and subsequent inadequate follow-up. An additional 19.3% of patients (110/569) had previously received ART, but had interrupted therapy. Among those currently on ART, nearly one half were not virologically suppressed, suggesting recent ART initiation, poor adherence, and/or ART resistance.

Strikingly, 25% of HIV-infected patients were on effective ART with a suppressed viral load and yet developed conditions requiring hospitalization. One explanation is that a substantial proportion of patients in resource-limited settings still commence ART with low CD4 counts^26^ and remain at high risk for opportunistic infections during early ART. For similar reasons, if patients start ART with low CD4 counts and later interrupt, the CD4 count drops to pre-ART levels rapidly putting them at high risk of infectious complications.^27^ Historically, in communities served by this hospital, patients started ART with very low CD4 counts.^28,29^ In the present study, the median current CD4 count in those who had interrupted ART was 71 cells/μL.

Our findings suggest that much of the contemporary hospital-level morbidity is not at the “front end” of the cascade (prior to HIV diagnosis and ART), but at the “back end” in patients failing virologically while on ART or disengaging from ART services – a different scenario to the years before and during early ART scale-up.^30–32^ One-fifth of HIV-related admissions were of patients who had interrupted ART; these patients had started ART a median of 4.7 years previously. A systematic review reported 29.5% median attrition from African ART clinics by 36 months, with 59% of this attrition attributed to loss to follow-up.^33^ Contributing factors may include patients’ perception of their health, life events, financial and occupation issues, and experiences at their clinic.^33–36^ As clinic patient numbers have grown, retaining patients in ART care has been challenging.^37,38^ A related problem is poor adherence on ART; if detection of virological failure is delayed, patients may experience clinical deterioration needing hospital admission prior to appropriate switch to second-line therapy. Some patients have ongoing adherence problems on second-line, remaining unsuppressed. Strategies to monitor and optimize adherence and retention are critical for maximizing ARTs public health benefit. “Adherence clubs” in Khayelitsha are a novel and promising strategy.^39,40^

An important reason for the differential effect of ART on hospitalization in this setting compared with well-resourced settings is the high prevalence of co-infections. TB contributed directly to 43.6% of admissions, and 37.2% of deaths within 90 days. Patients not on ART or on ART with low CD4 counts are particularly susceptible, but even in patients on ART in these communities, the risk of TB remains considerably elevated compared to that of HIV-negative people, being 4-fold higher even with CD4 counts >700 cells/μL on ART.^41^ In our study, 6.2% (n = 12) of patients with newly diagnosed TB had a CD4 count between 350 and 499 cells/μL, and a further 5.6% (n = 11) had a CD4 count ≥500 cells/μL. TB was also the commonest reason for admission in those with suppressed viral load. In a recent systematic review of post-mortem studies, TB was reported to account for approximately 40% of facility-based HIV-related adult deaths in resource-limited settings.^42^ AIDS-related conditions other than TB accounted for 1 in 10 admissions, yet were an independent predictor of mortality accounting for one-quarter of deaths.

What are the messages for health system planners from our findings? First, even with successful ART scale-up in sub-Saharan Africa as in the Western Cape province,^43^ HIV-related hospitalizations remain very common. Second, interventions to reduce hospitalizations need to focus on retention in care, adherence, and preventing active TB. ART reduces TB incidence by 65% in HIV-infected people,^44^ underlining the need to ensure optimal functioning ART programmes in high TB burden communities. Also, isoniazid preventive therapy in patients on ART reduces TB incidence by 37%.^45^ Comprehensive integration of ART and TB services is also a priority. Third, hospital-based surveillance provides an important indicator of the conditions contributing to morbidity and mortality in ART programmes and programme deficiencies. It provides similar information to monitoring causes of death at a broader level without the difficulties of determining mortality attribution in the community and with more accurate diagnostic information. With the WHO now advising ART for all HIV-infected people at diagnosis regardless of CD4 count,^46^ it can be anticipated that numbers of patients requiring ART will increase substantially in the next few years, adding to the challenges faced by ART services in addressing the “cracks” we identified.

Study strengths were the inclusion of an unselected cross-sectional cohort of patients to minimize selection bias, systematic screening for TB in all participants, and our ability to establish a diagnosis in almost all patients (99.5%). Vital status at 90 days was ascertained in approximately 95% of patients. There are limitations to our study. This was a single center study. However, we believe the results are generalizable to other inpatient settings in South Africa as this study was conducted at a district hospital similar to hospitals found across South Africa, and the HIV seroprevalence in surrounding communities is similar to national seroprevalence. However, ART coverage in Western Cape is high and in settings with lower coverage, hospital-related morbidity and mortality may well be even higher. The study was performed at a hospital level without robust data regarding the denominator population meaning we were unable to calculate reliable incidence rates for the broader HIV population. Some patients who reported not currently being on ART were virologically suppressed. This could be due to misclassification, laboratory technical issues, or clinical factors (recently interrupted ART or elite or post-treatment controllers). Patients admitted with known drug-resistant TB were typically admitted to separate isolation rooms and not enrolled.

In conclusion, despite widespread ART availability in surrounding communities, HIV still contributed to over 60% of medical admissions to this South African hospital. One-third of patients required hospital readmission and mortality approached 15% at 90 days. This is a substantial resource burden for overstretched health services. TB was the most common primary clinical diagnosis and leading cause of death. “Cracks” at points throughout the HIV care cascade were key drivers. Additional interventions are required within the HIV programme to reduce these admissions. Several such interventions have been proposed, and have or are being evaluated.^39,40,47–50^
